# Piperazine-1,4-diium bis­(pyridine-2,6-dicarboxyl­ato-κ^3^ *O* ^2^,*N*,*O* ^6^)cobaltate(II) tetra­hydrate

**DOI:** 10.1107/S1600536811023518

**Published:** 2011-06-25

**Authors:** Akbar Raissi Shabari, Nazanin Ghoddoosi, Mehrdad Pourayoubi, Shahram Moradi

**Affiliations:** a Faculty of Chemistry, North Tehran Branch, Islamic Azad University, Tehran, Iran; b Department of Chemistry, Ferdowsi University of Mashhad, Mashhad, 91779, Iran

## Abstract

The asymmetric unit of the title complex, (C_4_H_12_N_2_)[Co(C_7_H_3_NO_4_)_2_]·4H_2_O, consists of one piperazinediium dication, one [Co(py-2,6-dc)_2_]^2−^ dianion (where py-2,6-dc is pyridine-2,6-dicarboxyl­ate) and four water mol­ecules. The piperazinediium cation adopts a chair conformation and the Co^II^ ion is six-coordinated in an N_2_O_4_ environment, having a distorted octa­hedral geometry. In the crystal, inter­molecular O—H⋯O, N—H⋯O and weak C—H⋯O hydrogen bonds link the components, forming a three-dimensional network.

## Related literature

The structure determination of title compound was performed as a part of our project on the synthesis of new proton-transfer compounds, see: Raissi Shabari *et al.* (2010 ▶). For bond lengths in a related cobaltate(II) complex, see: Pasdar *et al.* (2011 ▶). For bond lengths and angles in the piperazinediium dication, see: Sutherland & Harrison (2009 ▶); Allen *et al.* (1995 ▶). For positive-charge-assisted hydrogen bonds, see: Gilli *et al.* (1994 ▶).
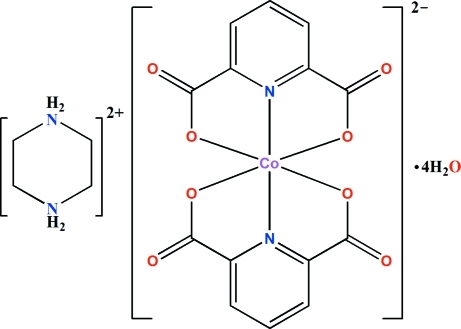


## Experimental

### 

#### Crystal data

(C_4_H_12_N_2_)[Co(C_7_H_3_NO_4_)_2_]·4H_2_O
*M* *_r_* = 549.36
Monoclinic, 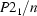

*a* = 7.9537 (16) Å
*b* = 13.420 (3) Å
*c* = 21.004 (4) Å
β = 90.55 (3)°
*V* = 2241.8 (8) Å^3^
*Z* = 4
Mo *K*α radiation
μ = 0.84 mm^−1^
*T* = 150 K
0.5 × 0.40 × 0.15 mm

#### Data collection

Stoe IPDS 2T diffractometer
Absorption correction: numerical [shape of crystal determined optically (*X-RED* and *X-SHAPE*; Stoe & Cie, 2005 ▶)] *T*_min_ = 0.670, *T*_max_ = 0.878
15339 measured reflections
6018 independent reflections
5226 reflections with *I* > 2σ(*I*)
*R*_int_ = 0.038

#### Refinement

*R*[*F* ^2^ > 2σ(*F* ^2^)] = 0.034
*wR*(*F* ^2^) = 0.077
*S* = 1.07
6018 reflections
364 parameters
H atoms treated by a mixture of independent and constrained refinement
Δρ_max_ = 0.48 e Å^−3^
Δρ_min_ = −0.33 e Å^−3^

### 

Data collection: *X-AREA* (Stoe & Cie, 2005 ▶); cell refinement: *X-AREA*; data reduction: *X-AREA*; program(s) used to solve structure: *SHELXS97* (Sheldrick, 2008 ▶); program(s) used to refine structure: *SHELXL97* (Sheldrick, 2008 ▶); molecular graphics: *ORTEP-3 for Windows* (Farrugia, 1997 ▶); software used to prepare material for publication: *WinGX* (Farrugia, 1999 ▶) and *enCIFer* (Allen *et al.*, 2004 ▶).

## Supplementary Material

Crystal structure: contains datablock(s) I, global. DOI: 10.1107/S1600536811023518/lh5266sup1.cif

Structure factors: contains datablock(s) I. DOI: 10.1107/S1600536811023518/lh5266Isup2.hkl

Additional supplementary materials:  crystallographic information; 3D view; checkCIF report

## Figures and Tables

**Table 1:** Hydrogen-bond geometry (Å, °)

*D*—H⋯*A*	*D*—H	H⋯*A*	*D*⋯*A*	*D*—H⋯*A*
O12—H12*B*⋯O3^i^	0.79 (3)	2.06 (3)	2.8428 (18)	174 (3)
O11—H11*B*⋯O5	0.83 (3)	2.00 (3)	2.8290 (18)	177 (3)
O11—H11*A*⋯O1^ii^	0.81 (3)	2.02 (3)	2.8173 (19)	170 (3)
O10—H10*B*⋯O7	0.79 (3)	2.15 (3)	2.9213 (19)	165 (3)
O10—H10*A*⋯O11^iii^	0.87 (3)	1.99 (3)	2.854 (2)	172 (3)
O9—H9*B*⋯O8	0.79 (3)	1.96 (3)	2.7521 (18)	175 (2)
O9—H9*A*⋯O12^iv^	0.83 (3)	2.01 (3)	2.840 (2)	173 (3)
N4—H4*B*⋯O3^v^	0.89 (2)	1.92 (2)	2.7973 (18)	165 (2)
N4—H4*A*⋯O6^iii^	0.91 (2)	1.89 (2)	2.7592 (17)	161 (2)
N3—H3*B*⋯O9^vi^	0.91 (2)	1.86 (2)	2.6958 (18)	152 (2)
N3—H3*A*⋯O2	0.91 (2)	2.50 (2)	3.1126 (19)	124.5 (18)
N3—H3*A*⋯O1	0.91 (2)	1.88 (2)	2.7957 (18)	176 (2)
C18—H18*B*⋯O10^vii^	0.97	2.58	3.457 (2)	151
C17—H17*B*⋯O7^vii^	0.97	2.36	3.127 (2)	135
C16—H16*B*⋯O12^iv^	0.97	2.52	3.293 (2)	137
C15—H15*B*⋯O10^vi^	0.97	2.60	3.261 (2)	126
C15—H15*A*⋯O2	0.97	2.54	3.140 (2)	120

Symmetry codes: (i) 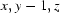; (ii) 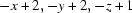; (iii) 
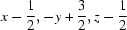
; (iv) 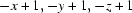; (v) 
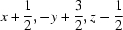
; (vi) 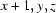; (vii) 
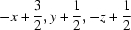
.

**Fig. 1.**
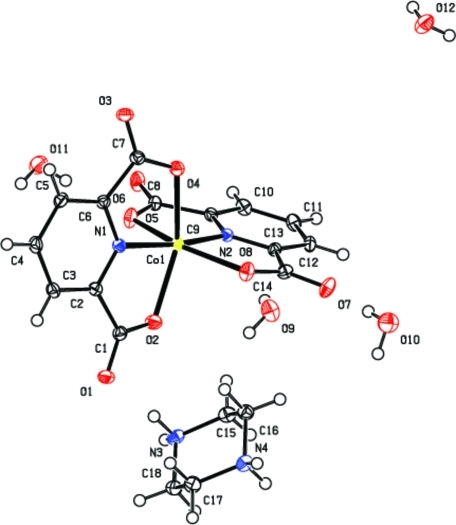
Molecular structure and atom labeling scheme for title complex with displacement ellipsoids drawn at the 50% probability level.

## supplementary crystallographic information

### Comment

The structure determination of title compound was performed as a part of a project on the synthesis of new proton-transfer compounds (Raissi Shabari *et al.*, 2010).

The Co atom in title compound adopts a distorted octahedral coordination arising from four oxygen and two nitrogen atoms of two pyridine-2,6-dicarboxylato ligands, Fig. 1. The Co—O and Co—N bond lengths are similar to those in a recently published bis(pyridine-2,6-dicarboxylato)cobaltate(II) complex, (C_6_H_10_N_2_)[Co(C_7_H_3_NO_4_)_2_].5H_2_O (Pasdar *et al.*, 2011). The organic dication adopts a typical chair geometry with normal bond lengths and angles (Sutherland & Harrison, 2009; Allen *et al.*, 1995).

The protonated piperazine nitrogen atoms are involved in positive charge assisted (Gilli *et al.*, 1994) N—H···O hydrogen bonds, atom N3 is involved with the O atoms of an adjacent carbonyl of one pyridine-2,6-dicarboxylato ligand and a neighbouring H_2_O molecule and atom N4 with two pyridine-2,6-dicarboxylato ligands in two neighbouring complexes.

Cations, anions and four crystallographically independent H_2_O molecules are hydrogen bonded in a three-dimensional network through N—H···O, O—H···O and weak C—H···O hydrogen bonds (Table 1).

### Experimental

A solution of Co(NO_3_)_2_.6H_2_O (2 mmol) in H_2_O was added to a solution of pyridine-2,6-dicarboxylic acid (4 mmol) in H_2_O and stirred (45 minutes) at room temperature. To the resulting solution, a solution of piperazine (4 mmol) in H_2_O was added and stirred (4 h) at 323 K. Suitable single crystals for X-ray analysis were obtained after slow evaporation at room temperature. IR (KBr, ν, cm^-1^): 3425, 3240, 3013, 3001, 2725, 2467, 1608, 1568, 1425, 1371, 1279, 1182, 1074, 1036, 914, 824, 760, 733, 692, 677, 592, 534.

### Refinement

H atoms of water molecules and N—H groups of cation were found in a difference Fourier map and refined isotropically. Carbon-bound H-atoms were placed in calculated positions, C—H = 0.93 Å (aromatic) and 0.97 Å (CH_2_), and were included in the refinement using a riding-model approximation, with *U*_iso_ = 1.2*U*_eq_ (C).

### Crystal data

**Table:**

(C_4_H_12_N_2_)[Co(C_7_H_3_NO_4_)_2_]·4H_2_O	*F*(000) = 1140
*M**_r_* = 549.36	*D*_x_ = 1.628 Mg m^−^^3^
Monoclinic, *P*2_1_/*n*	Mo *K*α radiation, λ = 0.71073 Å
Hall symbol: -P 2yn	Cell parameters from 6018 reflections
*a* = 7.9537 (16) Å	θ = 2.5–29.1°
*b* = 13.420 (3) Å	µ = 0.84 mm^−^^1^
*c* = 21.004 (4) Å	*T* = 150 K
β = 90.55 (3)°	Plate, pink
*V* = 2241.8 (8) Å^3^	0.5 × 0.4 × 0.15 mm
*Z* = 4	

### Data collection

**Table:**

Stoe IPDS 2T diffractometer	6018 independent reflections
Radiation source: fine-focus sealed tube	5226 reflections with *I* > 2σ(*I*)
graphite	*R*_int_ = 0.038
Detector resolution: 0.15 mm pixels mm^-1^	θ_max_ = 29.1°, θ_min_ = 2.5°
rotation method scans	*h* = −10→10
Absorption correction: numerical shape of crystal determined optically	*k* = −16→18
*T*_min_ = 0.670, *T*_max_ = 0.878	*l* = −24→28
15339 measured reflections	

### Refinement

**Table:**

Refinement on *F*^2^	Primary atom site location: structure-invariant direct methods
Least-squares matrix: full	Secondary atom site location: difference Fourier map
*R*[*F*^2^ > 2σ(*F*^2^)] = 0.034	Hydrogen site location: inferred from neighbouring sites
*wR*(*F*^2^) = 0.077	H atoms treated by a mixture of independent and constrained refinement
*S* = 1.07	*w* = 1/[σ^2^(*F*_o_^2^) + (0.0326*P*)^2^ + 1.2111*P*] where *P* = (*F*_o_^2^ + 2*F*_c_^2^)/3
6018 reflections	(Δ/σ)_max_ = 0.001
364 parameters	Δρ_max_ = 0.48 e Å^−^^3^
0 restraints	Δρ_min_ = −0.33 e Å^−^^3^

### Special details

**Table:**

Geometry. All e.s.d.'s (except the e.s.d. in the dihedral angle between two l.s. planes) are estimated using the full covariance matrix. The cell e.s.d.'s are taken into account individually in the estimation of e.s.d.'s in distances, angles and torsion angles; correlations between e.s.d.'s in cell parameters are only used when they are defined by crystal symmetry. An approximate (isotropic) treatment of cell e.s.d.'s is used for estimating e.s.d.'s involving l.s. planes.
Refinement. Refinement of *F*^2^ against ALL reflections. The weighted *R*-factor *wR* and goodness of fit *S* are based on *F*^2^, conventional *R*-factors *R* are based on *F*, with *F* set to zero for negative *F*^2^. The threshold expression of *F*^2^ > σ(*F*^2^) is used only for calculating *R*-factors(gt) *etc*. and is not relevant to the choice of reflections for refinement. *R*-factors based on *F*^2^ are statistically about twice as large as those based on *F*, and *R*- factors based on ALL data will be even larger.

### Fractional atomic coordinates and isotropic or equivalent isotropic displacement parameters (Å^2^)

**Table:**

	*x*	*y*	*z*	*U*_iso_*/*U*_eq_	
O10	0.26226 (16)	0.55638 (11)	0.28084 (7)	0.0288 (3)	
Co1	0.73056 (2)	0.745668 (14)	0.504511 (9)	0.01278 (6)	
C18	1.08242 (19)	0.81672 (13)	0.23202 (8)	0.0195 (3)	
H18A	1.1280	0.7659	0.2043	0.023*	
H18B	1.1624	0.8712	0.2344	0.023*	
C16	0.76380 (18)	0.73170 (12)	0.26866 (7)	0.0173 (3)	
H16A	0.6828	0.6777	0.2667	0.021*	
H16B	0.7185	0.7836	0.2957	0.021*	
C15	0.92728 (19)	0.69397 (11)	0.29675 (7)	0.0176 (3)	
H15A	0.9091	0.6714	0.3400	0.021*	
H15B	0.9670	0.6378	0.2720	0.021*	
C17	0.9172 (2)	0.85410 (12)	0.20509 (8)	0.0197 (3)	
H17A	0.8759	0.9083	0.2311	0.024*	
H17B	0.9339	0.8793	0.1623	0.024*	
N4	0.79141 (16)	0.77200 (10)	0.20359 (6)	0.0165 (2)	
N3	1.05640 (16)	0.77441 (11)	0.29680 (6)	0.0175 (3)	
O5	0.91680 (15)	0.73565 (8)	0.58111 (5)	0.0206 (2)	
N1	0.71594 (14)	0.89536 (9)	0.51050 (6)	0.0128 (2)	
O4	0.53764 (14)	0.77007 (8)	0.57578 (5)	0.0188 (2)	
O2	0.89349 (14)	0.79723 (8)	0.43015 (5)	0.0191 (2)	
O8	0.57909 (14)	0.68290 (8)	0.42961 (5)	0.0187 (2)	
C7	0.53098 (18)	0.85856 (11)	0.59591 (7)	0.0145 (3)	
C6	0.62806 (17)	0.93522 (11)	0.55788 (7)	0.0132 (3)	
C9	0.86203 (17)	0.56523 (11)	0.56247 (7)	0.0128 (3)	
C13	0.69018 (17)	0.53516 (11)	0.47424 (7)	0.0124 (3)	
O3	0.44917 (14)	0.88780 (9)	0.64298 (5)	0.0190 (2)	
O1	0.95932 (14)	0.93309 (8)	0.37446 (5)	0.0188 (2)	
O7	0.51331 (14)	0.53761 (9)	0.38257 (6)	0.0218 (2)	
N2	0.76472 (14)	0.59801 (9)	0.51494 (6)	0.0116 (2)	
C14	0.58397 (18)	0.58788 (11)	0.42379 (7)	0.0148 (3)	
C8	0.94256 (18)	0.64790 (11)	0.60154 (7)	0.0149 (3)	
C2	0.80213 (17)	0.95197 (11)	0.46993 (7)	0.0128 (3)	
C5	0.62615 (19)	1.03742 (11)	0.56736 (7)	0.0159 (3)	
H5	0.5664	1.0652	0.6008	0.019*	
C12	0.71303 (17)	0.43292 (11)	0.47966 (7)	0.0147 (3)	
H12	0.6622	0.3892	0.4510	0.018*	
C10	0.88885 (18)	0.46439 (11)	0.57169 (7)	0.0163 (3)	
H10	0.9551	0.4417	0.6054	0.020*	
C1	0.89276 (17)	0.89032 (11)	0.42059 (7)	0.0147 (3)	
C3	0.80328 (18)	1.05509 (11)	0.47560 (7)	0.0151 (3)	
H3	0.8610	1.0946	0.4468	0.018*	
C11	0.81438 (18)	0.39770 (11)	0.52927 (7)	0.0169 (3)	
H11	0.8322	0.3296	0.5340	0.020*	
C4	0.71541 (19)	1.09736 (11)	0.52577 (8)	0.0172 (3)	
H4	0.7165	1.1661	0.5315	0.021*	
O9	0.37881 (15)	0.76948 (10)	0.33746 (7)	0.0244 (3)	
O6	1.02757 (14)	0.62407 (9)	0.64909 (6)	0.0223 (2)	
O11	0.9680 (2)	0.87887 (11)	0.67821 (7)	0.0312 (3)	
O12	0.46115 (18)	0.06341 (10)	0.71858 (6)	0.0260 (3)	
H3B	1.157 (3)	0.7514 (16)	0.3122 (11)	0.029 (6)*	
H4B	0.828 (3)	0.7228 (18)	0.1787 (11)	0.029 (6)*	
H3A	1.022 (3)	0.8243 (18)	0.3232 (11)	0.031 (6)*	
H4A	0.694 (3)	0.7923 (19)	0.1846 (11)	0.034 (6)*	
H9B	0.437 (3)	0.7419 (18)	0.3625 (12)	0.031 (6)*	
H10B	0.324 (4)	0.561 (2)	0.3103 (15)	0.050 (8)*	
H10A	0.321 (3)	0.582 (2)	0.2501 (14)	0.051 (8)*	
H9A	0.432 (4)	0.815 (2)	0.3200 (14)	0.052 (8)*	
H12A	0.508 (3)	0.050 (2)	0.7517 (14)	0.051 (8)*	
H12B	0.464 (3)	0.013 (2)	0.6991 (14)	0.045 (8)*	
H11B	0.950 (3)	0.838 (2)	0.6492 (14)	0.046 (7)*	
H11A	0.989 (3)	0.929 (2)	0.6591 (13)	0.046 (7)*	

### Atomic displacement parameters (Å^2^)

**Table:**

	*U*^11^	*U*^22^	*U*^33^	*U*^12^	*U*^13^	*U*^23^
O10	0.0222 (6)	0.0391 (8)	0.0252 (7)	−0.0005 (5)	−0.0045 (5)	0.0017 (6)
Co1	0.01592 (9)	0.00943 (9)	0.01298 (10)	0.00047 (7)	−0.00079 (6)	0.00003 (7)
C18	0.0185 (7)	0.0244 (8)	0.0158 (7)	−0.0038 (6)	0.0021 (5)	−0.0007 (6)
C16	0.0154 (6)	0.0188 (7)	0.0177 (7)	0.0008 (5)	0.0014 (5)	−0.0008 (5)
C15	0.0211 (7)	0.0161 (7)	0.0156 (7)	0.0040 (5)	−0.0001 (5)	0.0027 (5)
C17	0.0262 (8)	0.0165 (7)	0.0165 (7)	−0.0008 (6)	−0.0016 (6)	0.0040 (6)
N4	0.0158 (6)	0.0190 (6)	0.0147 (6)	0.0039 (5)	−0.0044 (5)	0.0003 (5)
N3	0.0149 (6)	0.0247 (7)	0.0128 (6)	0.0044 (5)	−0.0024 (5)	−0.0024 (5)
O5	0.0275 (6)	0.0138 (5)	0.0202 (5)	−0.0027 (4)	−0.0086 (4)	−0.0010 (4)
N1	0.0126 (5)	0.0118 (5)	0.0140 (6)	0.0001 (4)	0.0003 (4)	0.0004 (4)
O4	0.0240 (5)	0.0130 (5)	0.0194 (5)	−0.0025 (4)	0.0056 (4)	−0.0011 (4)
O2	0.0243 (5)	0.0142 (5)	0.0188 (5)	0.0013 (4)	0.0063 (4)	−0.0001 (4)
O8	0.0235 (5)	0.0147 (5)	0.0177 (5)	0.0013 (4)	−0.0082 (4)	0.0011 (4)
C7	0.0156 (6)	0.0153 (7)	0.0126 (6)	−0.0003 (5)	−0.0003 (5)	0.0021 (5)
C6	0.0149 (6)	0.0127 (6)	0.0120 (6)	0.0004 (5)	0.0000 (5)	0.0002 (5)
C9	0.0113 (6)	0.0143 (6)	0.0129 (6)	−0.0016 (5)	−0.0012 (5)	0.0005 (5)
C13	0.0113 (6)	0.0139 (6)	0.0120 (6)	0.0000 (5)	−0.0007 (5)	−0.0011 (5)
O3	0.0236 (5)	0.0180 (5)	0.0154 (5)	−0.0002 (4)	0.0067 (4)	0.0003 (4)
O1	0.0230 (5)	0.0181 (5)	0.0154 (5)	−0.0018 (4)	0.0053 (4)	0.0013 (4)
O7	0.0243 (6)	0.0231 (6)	0.0179 (5)	0.0034 (4)	−0.0092 (4)	−0.0060 (4)
N2	0.0111 (5)	0.0121 (5)	0.0117 (5)	−0.0002 (4)	−0.0005 (4)	0.0004 (4)
C14	0.0140 (6)	0.0180 (7)	0.0123 (6)	0.0026 (5)	−0.0014 (5)	0.0002 (5)
C8	0.0137 (6)	0.0170 (7)	0.0139 (6)	−0.0036 (5)	−0.0013 (5)	−0.0015 (5)
C2	0.0120 (6)	0.0138 (6)	0.0124 (6)	0.0002 (5)	−0.0004 (5)	0.0012 (5)
C5	0.0191 (6)	0.0145 (7)	0.0141 (6)	0.0000 (5)	0.0005 (5)	−0.0031 (5)
C12	0.0143 (6)	0.0124 (6)	0.0173 (7)	−0.0019 (5)	−0.0009 (5)	−0.0031 (5)
C10	0.0152 (6)	0.0163 (7)	0.0174 (7)	0.0002 (5)	−0.0033 (5)	0.0045 (5)
C1	0.0133 (6)	0.0168 (7)	0.0142 (6)	−0.0007 (5)	−0.0003 (5)	−0.0014 (5)
C3	0.0160 (6)	0.0126 (6)	0.0168 (7)	−0.0020 (5)	−0.0004 (5)	0.0018 (5)
C11	0.0178 (6)	0.0109 (6)	0.0221 (7)	0.0002 (5)	−0.0009 (6)	0.0024 (5)
C4	0.0204 (7)	0.0109 (6)	0.0202 (7)	−0.0016 (5)	−0.0003 (6)	−0.0007 (5)
O9	0.0182 (5)	0.0233 (6)	0.0315 (7)	0.0007 (5)	−0.0089 (5)	0.0059 (5)
O6	0.0243 (5)	0.0227 (6)	0.0196 (6)	−0.0062 (4)	−0.0116 (4)	0.0031 (5)
O11	0.0541 (9)	0.0202 (6)	0.0195 (6)	−0.0074 (6)	0.0038 (6)	−0.0005 (5)
O12	0.0400 (7)	0.0204 (6)	0.0175 (6)	0.0034 (5)	−0.0031 (5)	−0.0012 (5)

### Geometric parameters (Å, °)

**Table:**

O10—H10B	0.79 (3)	O8—C14	1.2817 (19)
O10—H10A	0.87 (3)	C7—O3	1.2518 (19)
Co1—N2	2.0117 (13)	C7—C6	1.518 (2)
Co1—N1	2.0162 (13)	C6—C5	1.386 (2)
Co1—O8	2.1446 (12)	C9—N2	1.3321 (18)
Co1—O2	2.1532 (13)	C9—C10	1.383 (2)
Co1—O4	2.1787 (13)	C9—C8	1.518 (2)
Co1—O5	2.1807 (13)	C13—N2	1.3357 (18)
C18—N3	1.491 (2)	C13—C12	1.389 (2)
C18—C17	1.511 (2)	C13—C14	1.5229 (19)
C18—H18A	0.9700	O1—C1	1.2485 (19)
C18—H18B	0.9700	O7—C14	1.2293 (18)
C16—N4	1.488 (2)	C8—O6	1.2426 (18)
C16—C15	1.510 (2)	C2—C3	1.389 (2)
C16—H16A	0.9700	C2—C1	1.514 (2)
C16—H16B	0.9700	C5—C4	1.388 (2)
C15—N3	1.490 (2)	C5—H5	0.9300
C15—H15A	0.9700	C12—C11	1.394 (2)
C15—H15B	0.9700	C12—H12	0.9300
C17—N4	1.488 (2)	C10—C11	1.391 (2)
C17—H17A	0.9700	C10—H10	0.9300
C17—H17B	0.9700	C3—C4	1.391 (2)
N4—H4B	0.89 (2)	C3—H3	0.9300
N4—H4A	0.91 (2)	C11—H11	0.9300
N3—H3B	0.91 (2)	C4—H4	0.9300
N3—H3A	0.91 (2)	O9—H9B	0.79 (3)
O5—C8	1.2692 (19)	O9—H9A	0.83 (3)
N1—C6	1.3335 (19)	O11—H11B	0.83 (3)
N1—C2	1.3358 (19)	O11—H11A	0.81 (3)
O4—C7	1.2619 (18)	O12—H12A	0.80 (3)
O2—C1	1.2652 (19)	O12—H12B	0.79 (3)
			
H10B—O10—H10A	103 (3)	C6—N1—Co1	118.50 (10)
N2—Co1—N1	169.23 (5)	C2—N1—Co1	119.77 (10)
N2—Co1—O8	76.55 (5)	C7—O4—Co1	113.82 (10)
N1—Co1—O8	113.87 (5)	C1—O2—Co1	115.50 (10)
N2—Co1—O2	108.30 (5)	C14—O8—Co1	116.32 (9)
N1—Co1—O2	76.15 (5)	O3—C7—O4	125.69 (14)
O8—Co1—O2	86.10 (5)	O3—C7—C6	118.30 (13)
N2—Co1—O4	99.70 (5)	O4—C7—C6	115.99 (13)
N1—Co1—O4	76.46 (5)	N1—C6—C5	120.71 (14)
O8—Co1—O4	99.71 (5)	N1—C6—C7	113.19 (12)
O2—Co1—O4	151.99 (4)	C5—C6—C7	126.06 (13)
N2—Co1—O5	76.63 (4)	N2—C9—C10	121.04 (13)
N1—Co1—O5	93.13 (4)	N2—C9—C8	113.76 (13)
O8—Co1—O5	152.85 (4)	C10—C9—C8	125.15 (13)
O2—Co1—O5	98.38 (5)	N2—C13—C12	120.96 (13)
O4—Co1—O5	88.87 (5)	N2—C13—C14	113.07 (12)
N3—C18—C17	109.89 (13)	C12—C13—C14	125.97 (13)
N3—C18—H18A	109.7	C9—N2—C13	121.49 (13)
C17—C18—H18A	109.7	C9—N2—Co1	118.93 (10)
N3—C18—H18B	109.7	C13—N2—Co1	119.58 (10)
C17—C18—H18B	109.7	O7—C14—O8	126.80 (14)
H18A—C18—H18B	108.2	O7—C14—C13	118.81 (13)
N4—C16—C15	110.28 (12)	O8—C14—C13	114.39 (12)
N4—C16—H16A	109.6	O6—C8—O5	126.53 (14)
C15—C16—H16A	109.6	O6—C8—C9	118.02 (13)
N4—C16—H16B	109.6	O5—C8—C9	115.43 (13)
C15—C16—H16B	109.6	N1—C2—C3	120.99 (14)
H16A—C16—H16B	108.1	N1—C2—C1	112.12 (12)
N3—C15—C16	110.38 (12)	C3—C2—C1	126.89 (13)
N3—C15—H15A	109.6	C6—C5—C4	118.47 (14)
C16—C15—H15A	109.6	C6—C5—H5	120.8
N3—C15—H15B	109.6	C4—C5—H5	120.8
C16—C15—H15B	109.6	C13—C12—C11	118.08 (13)
H15A—C15—H15B	108.1	C13—C12—H12	121.0
N4—C17—C18	110.13 (13)	C11—C12—H12	121.0
N4—C17—H17A	109.6	C9—C10—C11	118.40 (13)
C18—C17—H17A	109.6	C9—C10—H10	120.8
N4—C17—H17B	109.6	C11—C10—H10	120.8
C18—C17—H17B	109.6	O1—C1—O2	125.15 (14)
H17A—C17—H17B	108.1	O1—C1—C2	119.23 (13)
C16—N4—C17	110.77 (12)	O2—C1—C2	115.62 (13)
C16—N4—H4B	108.8 (15)	C2—C3—C4	117.93 (14)
C17—N4—H4B	109.6 (15)	C2—C3—H3	121.0
C16—N4—H4A	112.4 (15)	C4—C3—H3	121.0
C17—N4—H4A	110.8 (16)	C10—C11—C12	120.02 (14)
H4B—N4—H4A	104 (2)	C10—C11—H11	120.0
C15—N3—C18	112.15 (12)	C12—C11—H11	120.0
C15—N3—H3B	110.8 (14)	C5—C4—C3	120.28 (14)
C18—N3—H3B	109.0 (14)	C5—C4—H4	119.9
C15—N3—H3A	108.6 (14)	C3—C4—H4	119.9
C18—N3—H3A	108.7 (15)	H9B—O9—H9A	110 (2)
H3B—N3—H3A	107 (2)	H11B—O11—H11A	103 (3)
C8—O5—Co1	114.39 (9)	H12A—O12—H12B	104 (3)
C6—N1—C2	121.59 (13)		
			
N4—C16—C15—N3	56.04 (16)	C8—C9—N2—Co1	−2.77 (16)
N3—C18—C17—N4	−57.15 (17)	C12—C13—N2—C9	−0.7 (2)
C15—C16—N4—C17	−58.43 (16)	C14—C13—N2—C9	179.69 (12)
C18—C17—N4—C16	59.08 (16)	C12—C13—N2—Co1	179.15 (11)
C16—C15—N3—C18	−55.72 (17)	C14—C13—N2—Co1	−0.48 (16)
C17—C18—N3—C15	56.17 (17)	N1—Co1—N2—C9	−12.6 (3)
N2—Co1—O5—C8	−8.50 (11)	O8—Co1—N2—C9	−178.50 (11)
N1—Co1—O5—C8	168.13 (11)	O2—Co1—N2—C9	100.29 (11)
O8—Co1—O5—C8	−17.59 (17)	O4—Co1—N2—C9	−80.74 (11)
O2—Co1—O5—C8	−115.43 (11)	O5—Co1—N2—C9	5.75 (10)
O4—Co1—O5—C8	91.75 (11)	N1—Co1—N2—C13	167.6 (2)
N2—Co1—N1—C6	−59.3 (3)	O8—Co1—N2—C13	1.66 (10)
O8—Co1—N1—C6	105.67 (11)	O2—Co1—N2—C13	−79.55 (11)
O2—Co1—N1—C6	−175.04 (11)	O4—Co1—N2—C13	99.42 (11)
O4—Co1—N1—C6	10.90 (10)	O5—Co1—N2—C13	−174.09 (11)
O5—Co1—N1—C6	−77.18 (11)	Co1—O8—C14—O7	−176.49 (13)
N2—Co1—N1—C2	116.5 (3)	Co1—O8—C14—C13	3.39 (16)
O8—Co1—N1—C2	−78.56 (11)	N2—C13—C14—O7	177.88 (14)
O2—Co1—N1—C2	0.73 (10)	C12—C13—C14—O7	−1.7 (2)
O4—Co1—N1—C2	−173.33 (11)	N2—C13—C14—O8	−2.00 (18)
O5—Co1—N1—C2	98.59 (11)	C12—C13—C14—O8	178.38 (14)
N2—Co1—O4—C7	156.74 (10)	Co1—O5—C8—O6	−171.44 (13)
N1—Co1—O4—C7	−12.98 (10)	Co1—O5—C8—C9	9.52 (16)
O8—Co1—O4—C7	−125.39 (10)	N2—C9—C8—O6	175.91 (13)
O2—Co1—O4—C7	−25.34 (16)	C10—C9—C8—O6	−6.8 (2)
O5—Co1—O4—C7	80.51 (11)	N2—C9—C8—O5	−4.96 (19)
N2—Co1—O2—C1	−176.16 (10)	C10—C9—C8—O5	172.35 (14)
N1—Co1—O2—C1	−6.37 (10)	C6—N1—C2—C3	−0.1 (2)
O8—Co1—O2—C1	109.39 (11)	Co1—N1—C2—C3	−175.70 (10)
O4—Co1—O2—C1	6.00 (17)	C6—N1—C2—C1	179.60 (12)
O5—Co1—O2—C1	−97.55 (11)	Co1—N1—C2—C1	3.96 (15)
N2—Co1—O8—C14	−2.85 (11)	N1—C6—C5—C4	1.1 (2)
N1—Co1—O8—C14	180.00 (10)	C7—C6—C5—C4	−176.42 (13)
O2—Co1—O8—C14	107.02 (11)	N2—C13—C12—C11	0.8 (2)
O4—Co1—O8—C14	−100.60 (11)	C14—C13—C12—C11	−179.66 (13)
O5—Co1—O8—C14	6.25 (17)	N2—C9—C10—C11	1.3 (2)
Co1—O4—C7—O3	−168.85 (12)	C8—C9—C10—C11	−175.84 (14)
Co1—O4—C7—C6	12.83 (16)	Co1—O2—C1—O1	−169.47 (12)
C2—N1—C6—C5	−1.3 (2)	Co1—O2—C1—C2	10.27 (16)
Co1—N1—C6—C5	174.37 (11)	N1—C2—C1—O1	170.25 (13)
C2—N1—C6—C7	176.50 (12)	C3—C2—C1—O1	−10.1 (2)
Co1—N1—C6—C7	−7.81 (15)	N1—C2—C1—O2	−9.50 (18)
O3—C7—C6—N1	177.41 (13)	C3—C2—C1—O2	170.14 (14)
O4—C7—C6—N1	−4.14 (18)	N1—C2—C3—C4	1.6 (2)
O3—C7—C6—C5	−4.9 (2)	C1—C2—C3—C4	−178.01 (13)
O4—C7—C6—C5	173.53 (14)	C9—C10—C11—C12	−1.2 (2)
C10—C9—N2—C13	−0.4 (2)	C13—C12—C11—C10	0.2 (2)
C8—C9—N2—C13	177.06 (12)	C6—C5—C4—C3	0.5 (2)
C10—C9—N2—Co1	179.80 (11)	C2—C3—C4—C5	−1.8 (2)

### Hydrogen-bond geometry (Å, °)

**Table:**

*D*—H···*A*	*D*—H	H···*A*	*D*···*A*	*D*—H···*A*
O12—H12B···O3^i^	0.79 (3)	2.06 (3)	2.8428 (18)	174 (3)
O11—H11B···O5	0.83 (3)	2.00 (3)	2.8290 (18)	177 (3)
O11—H11A···O1^ii^	0.81 (3)	2.02 (3)	2.8173 (19)	170 (3)
O10—H10B···O7	0.79 (3)	2.15 (3)	2.9213 (19)	165 (3)
O10—H10A···O11^iii^	0.87 (3)	1.99 (3)	2.854 (2)	172 (3)
O9—H9B···O8	0.79 (3)	1.96 (3)	2.7521 (18)	175 (2)
O9—H9A···O12^iv^	0.83 (3)	2.01 (3)	2.840 (2)	173 (3)
N4—H4B···O3^v^	0.89 (2)	1.92 (2)	2.7973 (18)	165 (2)
N4—H4A···O6^iii^	0.91 (2)	1.89 (2)	2.7592 (17)	161 (2)
N3—H3B···O9^vi^	0.91 (2)	1.86 (2)	2.6958 (18)	152 (2)
N3—H3A···O2	0.91 (2)	2.50 (2)	3.1126 (19)	124.5 (18)
N3—H3A···O1	0.91 (2)	1.88 (2)	2.7957 (18)	176 (2)
C18—H18B···O10^vii^	0.97	2.58	3.457 (2)	151.
C17—H17B···O7^vii^	0.97	2.36	3.127 (2)	135.
C16—H16B···O12^iv^	0.97	2.52	3.293 (2)	137.
C15—H15B···O10^vi^	0.97	2.60	3.261 (2)	126.
C15—H15A···O2	0.97	2.54	3.140 (2)	120.

Symmetry codes: (i) *x*, *y*−1, *z*; (ii) −*x*+2, −*y*+2, −*z*+1; (iii) *x*−1/2, −*y*+3/2, *z*−1/2; (iv) −*x*+1, −*y*+1, −*z*+1; (v) *x*+1/2, −*y*+3/2, *z*−1/2; (vi) *x*+1, *y*, *z*; (vii) −*x*+3/2, *y*+1/2, −*z*+1/2.
